# Endoscopic Versus Surgical Bariatric Interventions for Obesity: A Systematic Review and Network Meta-Analysis

**DOI:** 10.14740/jocmr6627

**Published:** 2026-08-26

**Authors:** Mohammad Abdelmoaty, Abdulla Mohammad Abdulla Muhanna, Radwa Mohammed Mahmoud Attia, Adel Abdelwahed Ibrahim, Sarah Saeed Alqahtani, Hind Saeed Aseeri, Rawan Shaya Alqahtani, Bshar Jamal, Mohammad Alshehri, Ali Mohammed Alkasi, Nesreen Ali Ogran, Khaled Emad Elsayed Barakat, Ali Said Ali Metwaly

**Affiliations:** a Department of General Surgery, Armed Forces Hospital Southern Region: Khamis Mushait, 'Asir Region, Saudi Arabia; b Department of General and Bariatric Surgery, MOH King Fahad Hospital, Jeddah, Saudi Arabia; c Department of General Surgery, Faculty of Medicine, Alazhar University, Cairo, Egypt; d Department of Surgical Oncology, Faculty of Medicine, Alazhar University, Cairo, Egypt; e Public Health, Riyadh Second Health Cluster, Riyadh, Saudi Arabia; f Faculty of Medicine, Al-Quds University, Jerusalem, Palestine; g Department of General Surgery, College of Medicine, King Khalid University, Abha, Saudi Arabia; h Surgical Oncology Unit, Surgery Department, Faculty of Medicine, Alexandria University, Alexandria, Egypt; i Precision Medicine, Faculty of Pharmacy, Alexandria University, Alexandria, Egypt

**Keywords:** Endoscopic sleeve gastroplasty, Laparoscopic sleeve gastrectomy, Network meta-analysis, Bariatric surgery, Endoscopic bariatric therapy, Obesity

## Abstract

**Background:**

Laparoscopic sleeve gastrectomy (LSG) is the gold standard surgical intervention for obesity; however, it is underutilized because of patient reluctance and perioperative risks. Endoscopic sleeve gastroplasty (ESG) is a promising, minimally invasive alternative; however, comprehensive data comparing its efficacy and safety with those of the broader spectrum of bariatric interventions remain fragmented. This study aimed to evaluate the comparative effectiveness and safety of endoscopic and surgical bariatric modalities through a systematic review and network meta-analysis (NMA).

**Methods:**

Major electronic databases (PubMed, Embase, Cochrane Central Register of Controlled Trials (CENTRAL), Scopus) were searched from January 2010 to April 2026. Studies directly comparing endoscopic bariatric therapies (ESGs, intragastric balloons (IGBs)) with surgical interventions (LSG, laparoscopic greater curve plication (LGCP), and laparoscopic adjustable gastric banding (LAGB)) or lifestyle modifications (high-intensity diet and lifestyle therapy (HIDLT)) were included. The primary outcomes were the percentage total body weight loss (%TBWL) at 12 months and the incidence of serious adverse events (SAEs). Frequentist random-effects pairwise and NMAs were performed. The certainty of evidence was appraised using the GRADE framework.

**Results:**

Seventeen studies (including matched cohorts and large national registries) met the inclusion criteria. In direct pairwise analysis, LSG achieved significantly greater 12-month %TBWL than ESG (mean differences (MD): –10.53%; 95% confidence interval (CI), –14.10 to –6.97, P < 0.0001). There was no statistically significant difference in SAEs between the ESG and LSG (risk ratio, 1.30; 95% CI, 0.97–1.73; P = 0.076). In the NMA, with LSG as the reference, all interventions yielded inferior weight loss: LGCP (MD: –2.18%), ESG (–11.33%), LAGB (–15.81%), HIDLT (–17.63%), and IGB (–19.00%). The treatment hierarchy rankings (P-scores) identified LSG as the most effective intervention (0.988), followed by LGCP (0.812) and ESG (0.600). The IGB ranked the lowest (0.089). Local node splitting revealed no significant inconsistencies between direct and indirect evidence.

**Conclusions:**

LSG maintains definitive superiority in 12-month weight-loss efficacy. However, ESG has demonstrated robust clinical effectiveness, significantly outperforming legacy endoscopic devices (IGBs), and lifestyle interventions. Although data cannot confirm that ESG is as safe as LSG, it remains an anatomy-preserving option with a lower risk of reflux, making it an excellent alternative for patients averse to or ineligible for conventional bariatric surgery.

## Introduction

Obesity is a chronic, progressive, and multifactorial disease that has reached epidemic proportions and carries a global burden of cardiovascular and metabolic morbidity [1]. Bariatric surgery, notably conventional laparoscopic sleeve gastrectomy (LSG) and Roux-en-Y gastric bypass (RYGB), is the gold standard for achieving substantial and durable weight loss and reversing obesity-related comorbidities [2]. Despite its proven clinical efficacy and long-term economic benefits, bariatric surgery is underutilized, with fewer than 1% of eligible patients undergoing surgical intervention annually. This treatment gap is driven by patient reluctance, high healthcare costs, anatomic irreversibility, and concerns regarding perioperative surgical risks [1, 2].

To address this critical therapeutic void, minimally invasive endoscopic bariatric therapies (EBTs) have emerged as a paradigm-shifting alternative [1]. Procedures such as endoscopic sleeve gastroplasty (ESG) and space-occupying devices, such as intragastric balloons (IGB), offer an anatomically preserved outpatient approach to weight management [1, 3, 4]. While the utilization of IGBs has shown fluctuating trends and variable long-term efficacy [3], ESG has gained significant global traction. By inducing meaningful weight loss through full-thickness gastric remodeling and delayed gastric emptying, ESG serves as a highly effective and less invasive intervention [1, 2]. Furthermore, procedures such as IGB insertion are occasionally utilized as precursors or adjuncts to subsequent surgical options, although their distinct impact on gastric volumetry and hormonal profiles compared to primary surgical interventions remains an area of ongoing investigation [4].

Recent literature has focused on comparing these emerging endoscopic techniques with standard surgical approaches. Several systematic reviews and meta-analyses comparing ESG to LSG have demonstrated that while LSG yields superior long-term total body weight loss (TBWL) and more robust metabolic control, ESG is associated with a favorable safety profile [5–7]. Specifically, ESG demonstrates a significantly lower incidence of serious adverse events (SAEs), shorter procedural times, and reduced hospital length of stay compared to its surgical counterpart [5, 8–10]. Moreover, ESG mitigates specific surgical complications, avoiding the high rates of *de novo* gastroesophageal reflux disease (GERD) observed after LSG [7, 8].

Despite this accumulating body of evidence, the literature remains fragmented, as most meta-analyses have been restricted to direct pairwise comparisons (e.g., ESG vs. LSG) and fail to broadly evaluate both endoscopic (ESG, IGB, aspiration therapy) and surgical (LSG, RYGB) interventions [2, 5, 9]. Furthermore, heterogeneous study designs, varying follow-up durations, and inconsistent reporting of cardiometabolic outcomes underscore the need for synthesis [6, 9]. Therefore, we conducted a systematic review and network meta-analysis (NMA) to compare the effectiveness, safety profiles, and comorbidity resolution of various endoscopic and surgical bariatric interventions. This study aimed to delineate therapeutic hierarchies across modalities and provide robust, evidence-based guidance for individualized management of obesity.

## Methods

### Study design and protocol registration

This systematic review and NMA were conducted in adherence to the Preferred Reporting Items for Systematic Reviews and Meta-Analyses (PRISMA) 2020 statement [11] and the PRISMA extension for Network Meta-Analyses (PRISMA-NMA) [12] (Supplementary Material 1, jocmr.elmerjournals.com). A *priori* study protocol was registered in the International Prospective Register of Systematic Reviews (PROSPERO; CRD420261336494) [13].

### Search strategy

A systematic literature search was conducted across major electronic databases, including PubMed/MEDLINE, Embase, Cochrane Central Register of Controlled Trials (CENTRAL), Scopus, and Web of Science, from January 2010 to April 2026. The search syntax was constructed using a combination of medical subject headings (MeSH), Emtree terms, and free-text keywords (with appropriate truncation and wildcards) tailored to each database. The search terms included, but were not limited to, “bariatric surgery,” “endoscopic bariatric therapy,” “endoscopic sleeve gastroplasty,” “intragastric balloon,” “laparoscopic sleeve gastrectomy,” and “Roux-en-Y gastric bypass.” Boolean operators (AND, OR, NOT) were strategically employed to combine the concepts of obesity, EBTs, and surgical interventions. To mitigate publication bias, gray literature was explored via ClinicalTrials.gov, the World Health Organization International Clinical Trials Registry Platform (WHO-ICTRP), and ProQuest. Cross-referencing (forward and backward citation searching) of the included studies and relevant systematic reviews was performed to ensure literature saturation.

### Eligibility criteria

Studies were selected based on the following predetermined Population, Intervention, Comparator, Outcomes (PICO) criteria: the population was adults (≥ 18 years) with obesity, defined as a body mass index (BMI) ≥ 30 kg/m^2^, with or without obesity-related cardiometabolic comorbidities; the interventions were minimally invasive EBTs, specifically ESG, fluid- or gas-filled IGB, and aspiration therapy; the comparators were conventional laparoscopic bariatric surgery, including LSG, RYGB, and intensive medical/lifestyle management; the primary efficacy outcomes were the magnitude of weight loss evaluated by the percentage of total body weight loss (%TBWL), percentage of excess weight loss (%EWL), and absolute BMI reduction. The primary safety outcome was the incidence of SAEs defined as Clavien-Dindo Classification grade ≥ III, while secondary outcomes included cardiometabolic comorbidity remission (type 2 diabetes, hypertension, dyslipidemia), readmission rates, and re-intervention rates; randomized controlled trials (RCTs) were also included, and high-quality nonrandomized studies (prospective and retrospective cohorts, and registry-based analyses) were also included provided they enrolled ≥ 100 participants and reported a minimum follow-up of 12 months for weight-loss outcomes.

Although this review defined obesity as a BMI ≥ 30 kg/m^2^, the eligibility thresholds applied within the included cohorts were not uniform across modalities. The surgical arms recruited patients meeting conventional bariatric indications, a BMI ≥ 40 kg/m^2^, or ≥ 35 kg/m^2^ in the presence of at least one obesity-related comorbidity, whereas endoscopic and lifestyle interventions were offered to patients with class I–II obesity (BMI 30 to < 40 kg/m^2^), including individuals who did not meet, or who declined, conventional surgical criteria. This divergence in operative thresholds partly underlies the between-arm variation in baseline BMI.

### Study selection and data extraction

Two investigators independently screened the records by title and abstract, followed by a full-text evaluation. Inter-rater reliability (IRR) for study inclusion was quantified using Cohen’s kappa coefficient (κ) [14], resolving discrepancies through adjudication by a third investigator. Data extraction was performed independently using standardized, pre-piloted, electronic forms. The extracted domains included study characteristics, patient demographics, procedural details, and clinical outcomes.

### Quality assessment and risk of bias (RoB)

The methodological quality and RoB were independently evaluated by two investigators. Nonrandomized and observational studies were assessed using the Risk of Bias in Nonrandomized Studies of Interventions (ROBINS-I) tool [15], categorizing the risk of confounding, selection, and measurement biases.

### Statistical analysis and pooling models

All statistical analyses were performed using R software (version 4.5.2) [16] with the meta, netmeta, and gemtc packages [17].

#### Pairwise meta-analysis

For direct comparisons, continuous outcomes were pooled using mean differences (MD) with 95% confidence intervals (CIs). Dichotomous outcomes (e.g., SAEs and comorbidity remission) were pooled using risk ratios (RRs) with 95% CIs. Given the anticipated clinical and methodological heterogeneity, a random-effects (RE) model utilizing the DerSimonian–Laird (DL) estimator was employed [18].

#### NMA

A frequentist NMA was constructed to synthesize direct and indirect evidence across multiple treatment modalities. The network geometry was visualized using network plots. Treatment hierarchies were established by calculating the surface under the cumulative ranking curve (SUCRA) probabilities, ranging from 0 (worst) to 1 (best) [19].

#### Heterogeneity and inconsistency

Statistical heterogeneity was quantified using Cochran’s Q test, the I^2^ statistic (with > 75% denoting substantial heterogeneity), and the τ^2^ (tau-squared) variance estimator [20]. To evaluate network transitivity and inconsistency, the node-splitting method was applied to assess local inconsistency between direct and indirect estimates, whereas the design-by-treatment interaction model was used to evaluate global inconsistency [21].

### Trial sequential analysis (TSA)

TSA was performed to evaluate the robustness of the pooled estimates and prevent type I and type II errors due to sparse data or repeated significance testing. TSA boundaries were constructed to determine whether the cumulative sample size surpassed the required information size for critical outcomes [22].

### Publication bias and certainty of evidence

Small-study effects and potential publication bias were visually assessed using contour-enhanced funnel plots and statistically quantified using Egger’s regression test (for continuous outcomes) and Harbord’s test (for dichotomous outcomes) [23]. The overall certainty of the evidence for each network estimate was critically appraised using the Grading of Recommendations Assessment, Development, and Evaluation (GRADE) framework, which evaluates the RoB, inconsistency, indirectness, imprecision, and publication bias [24].

### Ethical compliance

As this study was a systematic review and network meta-analysis based entirely on previously published literature, it did not involve new research on human or animal subjects. Therefore, neither ethical approval nor informed consent was required, and an ethical compliance statement is not applicable. The protocol was registered with PROSPERO (CRD420261336494).

## Results

### Study selection and baseline characteristics

**Figure 1:**
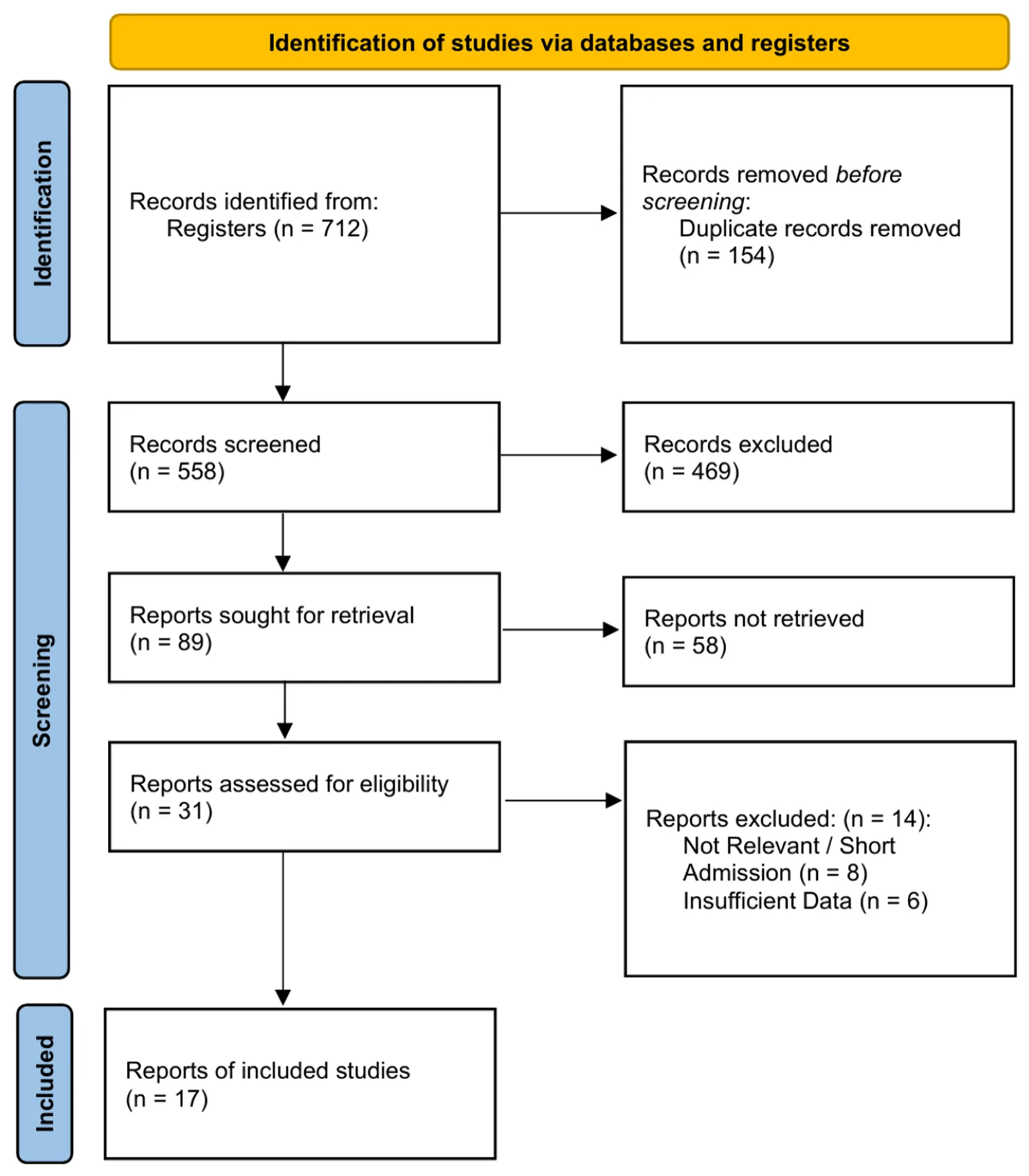
PRISMA 2020 flow diagram. PRISMA: Preferred Reporting Items for Systematic Reviews and Meta-Analyses.

The systematic literature search and study selection processes are detailed in the PRISMA flowchart (Fig. 1). A total of 17 studies met the pre-specified inclusion criteria, comprising retrospective cohorts, prospective matched cohorts, and large-scale registry analyses (e.g., Metabolic and Bariatric Surgery Accreditation and Quality Improvement Program (MBSAQIP) and TriNetX). In total, the network included data comparing ESG, LSG, laparoscopic adjustable gastric banding (LAGB), laparoscopic greater curve plication (LGCP), IGB, and high-intensity diet and lifestyle therapy (HIDLT) (Table 1) [25–41]. Excellent IRR was observed during the study screening phase (Cohen’s κ = 0.80, P < 0.001), indicating a substantial agreement between independent reviewers.

**Table 1:** Study Characteristics

Study	Study design	Treatment arms	Number
Gudur et al, 2023 [25]	Retrospective database (MBSAQIP)	ESG	6,054
		LSG	597,463
Carr et al, 2022 [26]	Prospective cohort	ESG	16
		LSG	45
Joseph et al, 2024 [27]	Retrospective cohort	ESG	68
		LSG	212
Dankar et al, 2025 [28]	Retrospective cohort	ESG	70
		LSG	70
Alqahtani et al, 2022 [29]	Propensity-matched cohort	ESG	3,018
		LSG	3,018
Fayad et al, 2019 [30]	Case-matched cohort	ESG	54
		LSG	83
Tammo et al, 2025 [31]	Retrospective cohort	EIB	36
		LSG	36
Lopez-Nava et al, 2021 [32]	Retrospective cohort	ESG	199
		LSG	61
		LGCP	36
Novikov et al, 2018 [33]	Retrospective cohort	ESG	91
		LSG	120
		LAGB	67
Marshall et al, 2022 [34]	Matched cohort	ESG	25
		LSG	25
Fiorillo et al, 2020 [35]	Propensity-matched cohort	ESG	23
		LSG	23
Mocanu et al, 2025 [36]	Retrospective database (MBSAQIP)	ESG	2,285
		LSG	504,312
Fayad et al, 2022 [37]	Propensity-matched database	EBT	211
		LBT	9,059
Osman et al, 2025 [38]	Propensity-matched cohort (TriNetX)	ESG	62
		LSG	62
Ebadinejad et al, 2025 [39]	Retrospective database (MBSAQIP)	ESG	2,171
		LSG	7,997
Milone et al, 2005 [40]	Retrospective cohort	BIB	57
		LSG	20
Cheskin et al, 2020 [41]	Case-matched cohort	ESG	105
		HIDLT	281

ESG: Endoscopic sleeve gastroplasty; LSG: Laparoscopic sleeve gastrectomy; EIB: endoscopic intragastric balloon; LGCP: Laparoscopic greater curve plication; LAGB: Laparoscopic adjustable gastric banding; EBT: endoscopic bariatric therapy; LBT: laparoscopic bariatric therapy; HIDLT: High-intensity diet and lifestyle therapy; BIB: BioEnterics intragastric balloon; MBSAQIP: Metabolic and Bariatric Surgery Accreditation and Quality Improvement Program.

Baseline BMI varied across studies and differed between treatment arms in the unmatched cohorts. Patients selected for endoscopic therapy presented with a lower baseline BMI than those undergoing LSG: 38.6 ± 7.0 vs. 47.2 ± 7.8 kg/m^2^ in Novikov et al [33], 39.6 ± 7.5 vs. 44.9 ± 7.7 kg/m^2^ in Mocanu et al [36], and 35.5 ± 5.2 vs. 40.7 ± 5.6 kg/m^2^ in Carr et al [26] (all P < 0.001); Marshall et al [34] reported a comparable gradient (median 33.4 vs. 39.6 kg/m^2^; P < 0.001), with a residual difference of 2.4 kg/m^2^ persisting after matching (P = 0.02), and Tammo et al [31] reported a significantly higher pre-procedural BMI in the LSG than in the balloon arm (P < 0.001). The matched analyses achieved balance between arms, with BMI values of 43.1 vs. 44.1 kg/m^2^ in Fayad et al [30] (P = 0.44), 39.4 ± 5.4 vs. 40.1 ± 3.7 kg/m^2^ in Lopez-Nava et al [32] (P = 0.42), 39.5 vs. 41.0 kg/m^2^ in Fiorillo et al [35] (P = 0.30), and 37.5 ± 1.4 vs. 37.6 ± 1.4 kg/m^2^ in Fayad et al [37] (P = 0.23), although these samples were comparatively small. Cheskin et al [41] matched ESG (40.5 ± 7.9 kg/m^2^) to lifestyle therapy (39.9 ± 7.6 kg/m^2^; P = 0.47), while Milone et al [40] was restricted to super-obesity (LSG 68.9 vs. balloon 58.4–60.2 kg/m^2^). Spanning more than 1 million patients, these data indicate that endoscopic therapy has been preferentially offered to patients with lower-grade obesity outside matched designs, a key source of residual confounding when interpreting the between-group weight-loss estimates, reflected in the ROBINS-I confounding domain (D1).

### RoB and certainty of evidence

**Figure 2:**
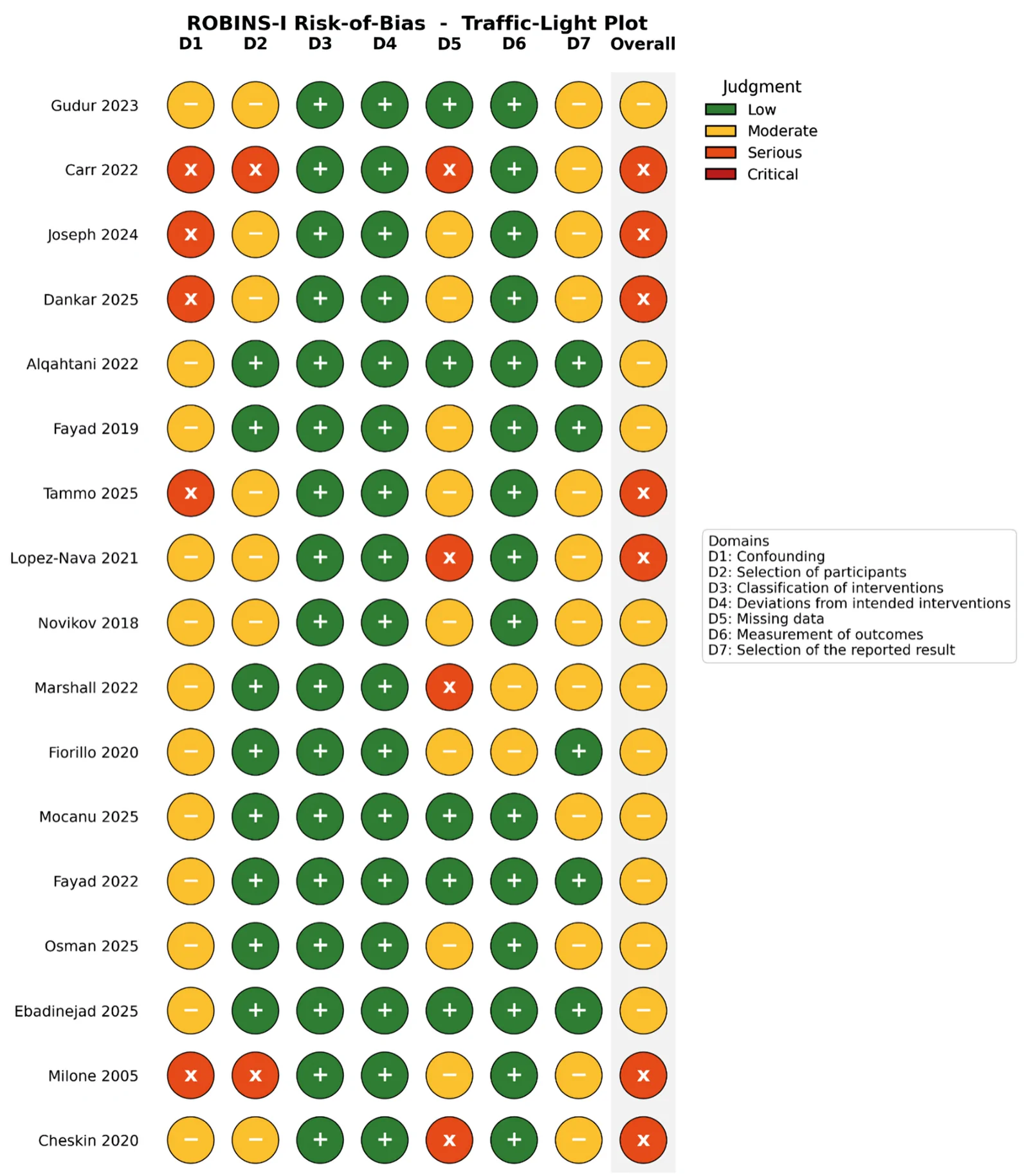
Risk of bias assessment using the ROBINS-I tool for the included nonrandomized studies (traffic-light plot of individual study domains). ROBINS-I: Risk of Bias in Nonrandomized Studies of Interventions.

**Figure 3:**
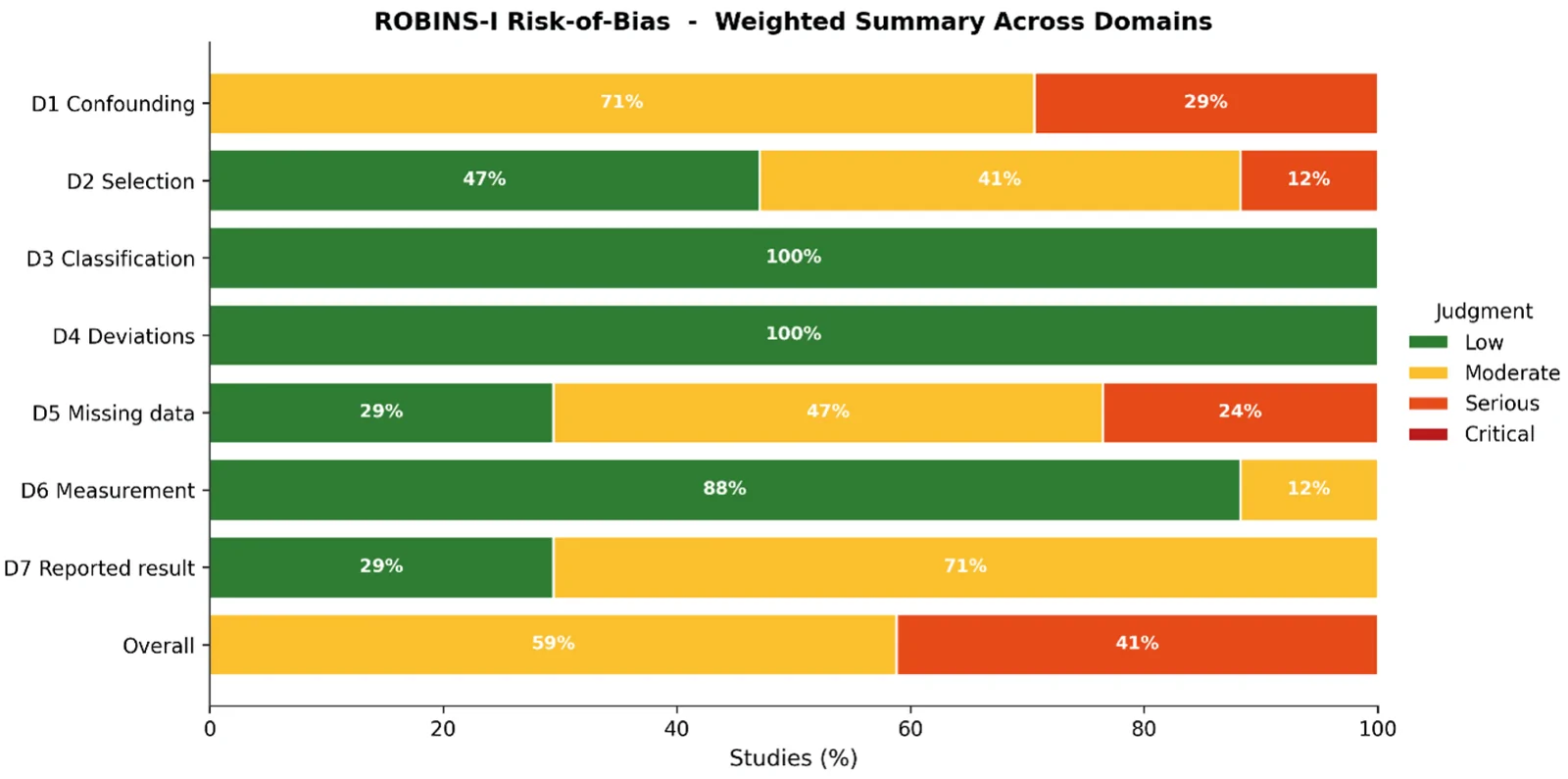
Risk of bias assessment using a weighted summary bar plot across all domains.

The RoB for all included nonrandomized studies was assessed using the ROBINS-I tool (Figs. 2, 3). The overall RoB was predominantly rated as moderate to serious. The primary methodological limitations across the evidence base were residual confounding due to a lack of randomization (D1) and missing data/attrition bias at the 12-month follow-up time points (D5). Using the GRADE framework, the overall certainty of evidence for all outcomes was very low, driven by the observational nature of the studies, high statistical heterogeneity, and imprecision stemming from small sample sizes in the non-registry cohorts (Table 2).

**Table 2:** GRADE Summary of Findings

Outcome	Studies (n)	Participants (n)	Effect estimate	Risk of bias	Inconsistency	Indirectness	Imprecision	Publication bias	Final certainty
%TBWL at 12 months (weight-loss efficacy)	8 cohorts	About 620,000	MD = –10.5 %TBWL (95% CI, –14.1 to –7.0) ESG vs LSG	Serious (–1)	Serious (–1)	Not serious	Not serious	Undetected	⊕◯◯◯ Very low
Serious adverse events (Clavien-Dindo ≥ III)	5 (including registries)	About 620,000	RR = 1.30 (95% CI, 0.97 to 1.73) ESG vs LSG	Serious (–1)	Not serious	Not serious	Serious (–1)	Undetected	⊕◯◯◯ Very low
Comorbidity remission (T2DM, GERD)	4 cohorts	About 6,200	T2DM remission: ESG 64% vs LSG 82%; New-onset GERD: ESG 4% vs LSG 20%	Serious (–1)	Serious (–1)	Not serious	Serious (–1)	Undetected	⊕◯◯◯ Very low

GRADE: Grading of Recommendations Assessment, Development, and Evaluation; MD: mean difference; RR: risk ratio; %TBWL: percentage of total body weight loss; CI: confidence interval; GERD: gastroesophageal reflux disease; T2DM: type 2 diabetes mellitus; ESG: endoscopic sleeve gastroplasty; LSG: laparoscopic sleeve gastrectomy.

### Efficacy and safety (ESG vs. LSG)

**Figure 4:**
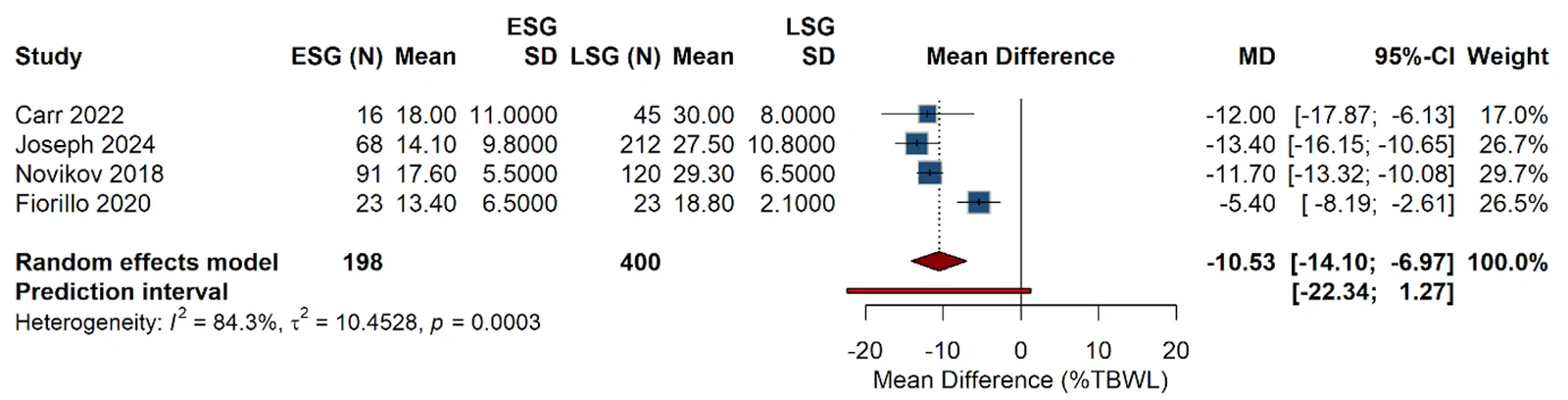
Pairwise meta-analysis forest plot showing the mean difference (MD) in percentage of total body weight loss (%TBWL) at 12 months (ESG vs. LSG). CI: confidence interval; ESG: endoscopic sleeve gastroplasty; LSG: laparoscopic sleeve gastrectomy; SD: standard deviation.

A direct pairwise meta-analysis was performed to compare ESG with the surgical gold standard, LSG. Regarding weight loss efficacy at 12 months, LSG demonstrated a significantly greater %TBWL than ESG (MD: –10.53%; 95% CI, –14.10 to –6.97, P < 0.0001) (Fig. 4). High statistical heterogeneity was observed for this continuous outcome (I^2^ = 84.3%, τ^2^ = 10.45, P = 0.0003).

**Figure 5:**
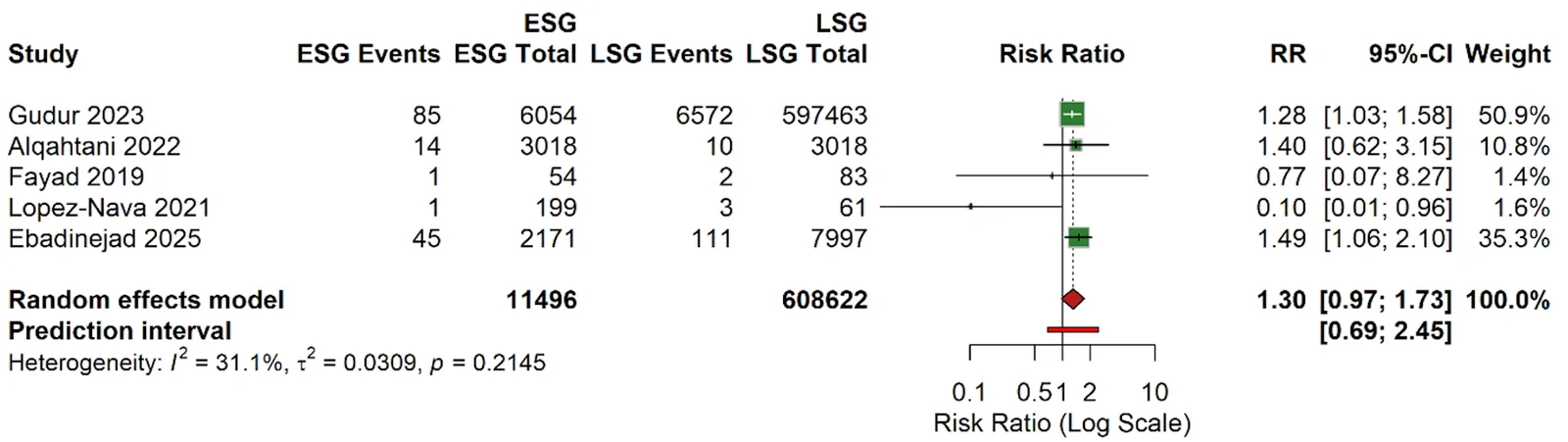
Pairwise meta-analysis forest plot showing the risk ratio (RR) of serious adverse events (SAEs, ESG vs. LSG). CI: confidence interval; ESG: endoscopic sleeve gastroplasty; LSG: laparoscopic sleeve gastrectomy.

Regarding the primary safety outcome, ESG was associated with a trend toward a higher incidence of SAEs (Clavien-Dindo ≥ III) than LSG, although this did not reach statistical significance in the random-effects model (RR, 1.30; 95% CI, 0.97–1.73; P = 0.076) (Fig. 5). Heterogeneity for the safety outcome was low to moderate (I^2^ = 31.1%, τ^2^ = 0.03, P = 0.21).

### Network geometry and relative efficacy

**Figure 6:**
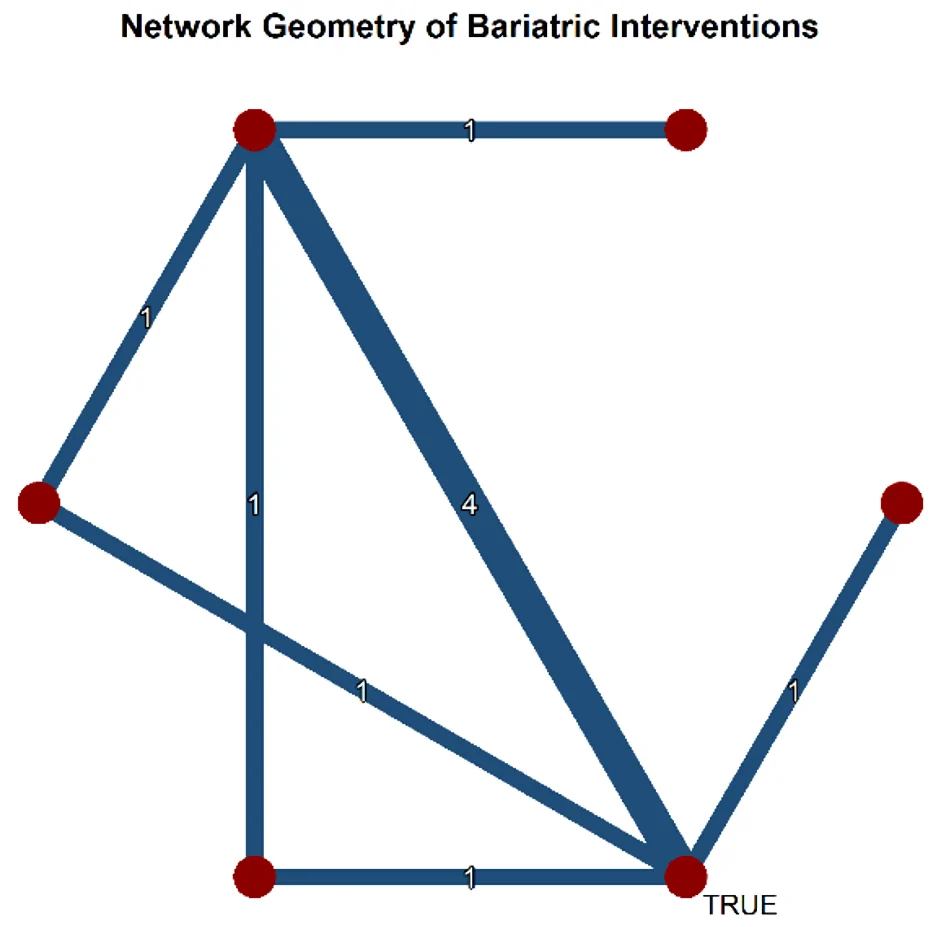
Network geometry graph of bariatric interventions. Node size is proportional to the total number of patients, and edge thickness represents the number of direct comparative studies.

A frequentist RE NMA was constructed to evaluate the comparative effectiveness of all bariatric modalities simultaneously. The network geometry (Fig. 6) illustrates that the ESG versus LSG comparison formed the most robust direct evidence link (greatest edge thickness), whereas interventions such as the IGB and HIDLT were connected to the network through single comparative nodes.

**Figure 7:**
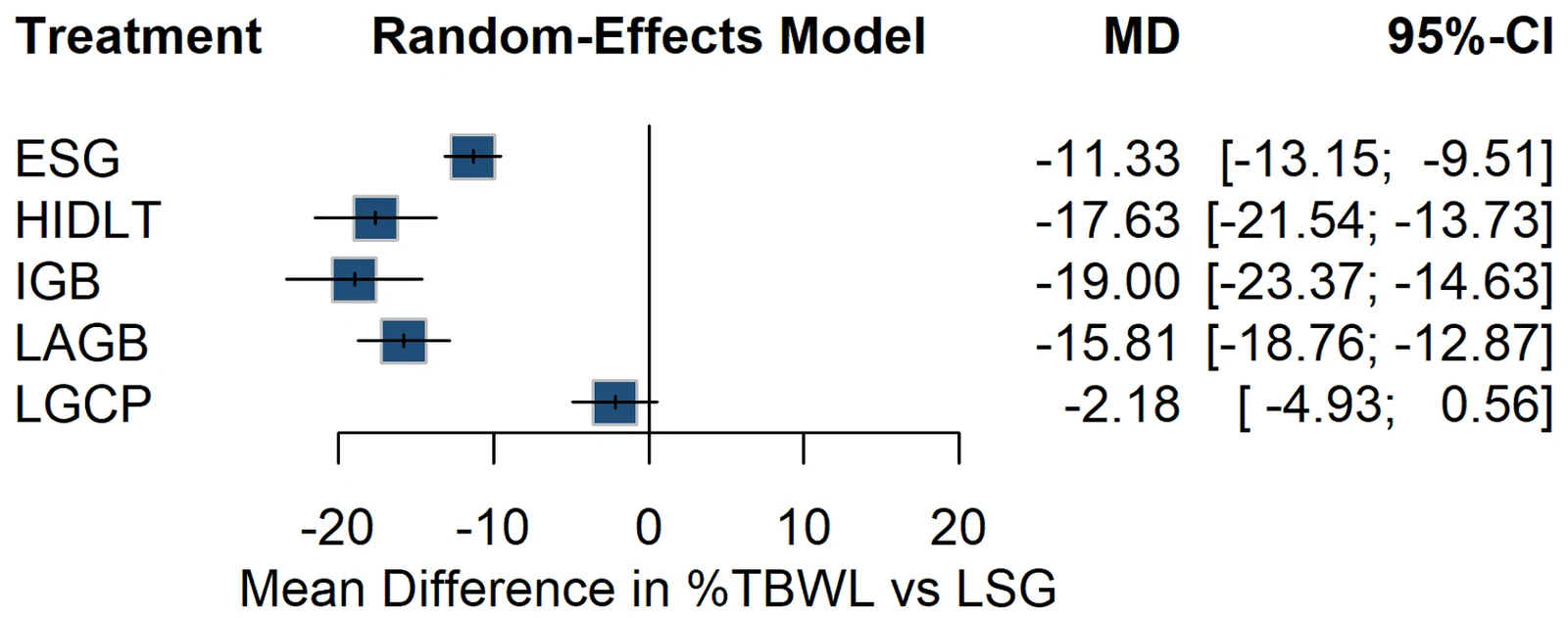
Network meta-analysis forest plot. The network estimates (MD; 95% CI) compared to LSG were as follows: LGCP (–2.18%; –4.93 to 0.56, P = 0.118), ESG (–11.33%; –13.15 to –9.51, P < 0.0001), LAGB (–15.81%; –18.76 to –12.87, P < 0.0001), HIDLT (–17.63%; –21.54 to –13.73, P < 0.0001), and IGB (–19.00%; –23.37 to –14.63, P < 0.0001). ESG: endoscopic sleeve gastroplasty; HIDLT: high-intensity diet and lifestyle therapy; IGB: intragastric balloon; LAGB: laparoscopic adjustable gastric banding; LGCP: laparoscopic greater curve plication; LSG: laparoscopic sleeve gastrectomy; %TBWL: percentage of total body weight loss; MD: mean difference; CI: confidence interval.

Setting LSG as the reference standard, the NMA revealed that all other interventions yielded significantly lower %TBWL at 12 months (Fig. 7).

### Treatment hierarchies (SUCRA rankings)

**Figure 8:**
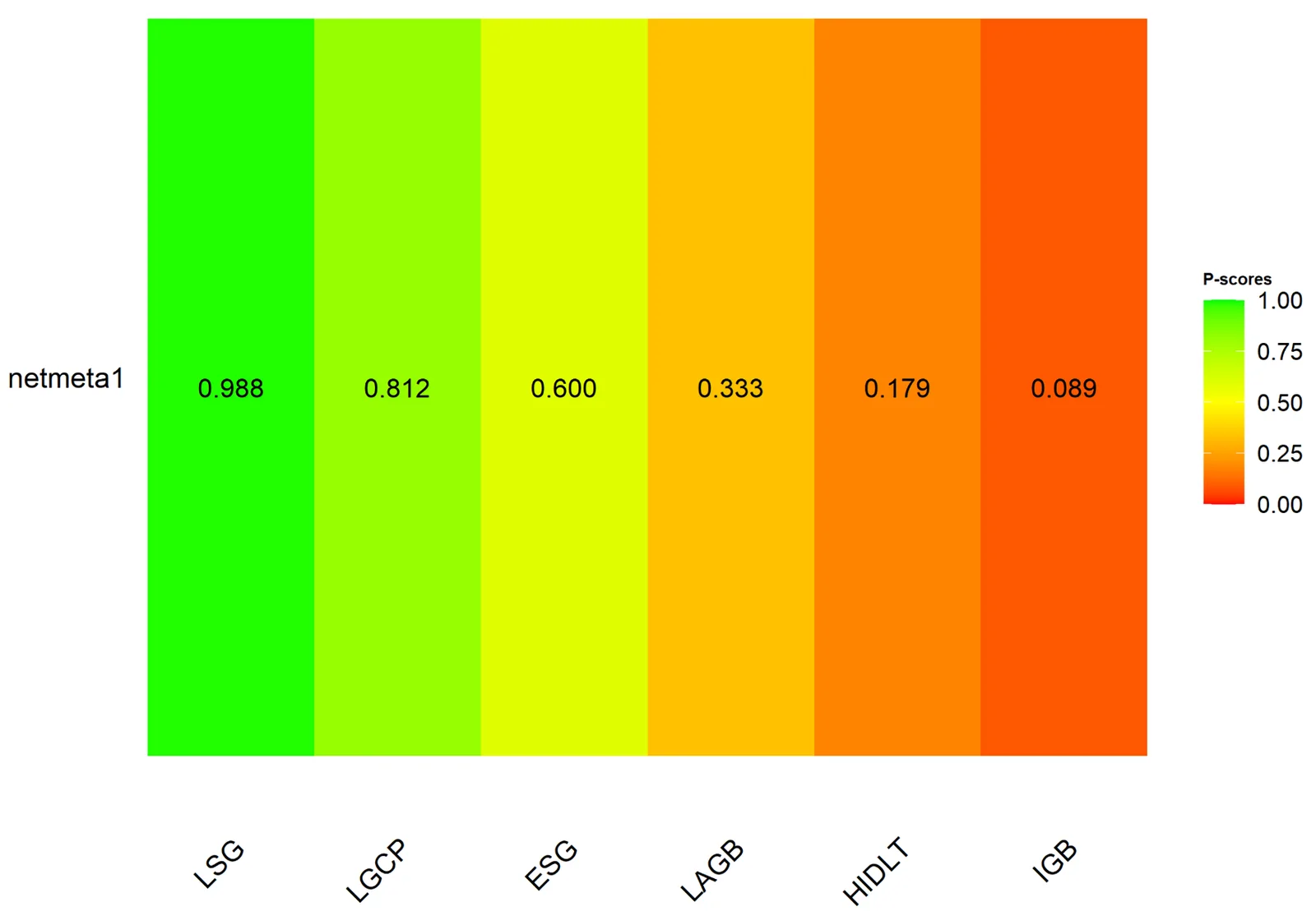
Treatment ranking heatmap (P-scores/SUCRA). A higher score (closer to 1.0) indicates a greater probability of the intervention yielding the highest %TBWL. ESG: endoscopic sleeve gastroplasty; HIDLT: high-intensity diet and lifestyle therapy; IGB: intragastric balloon; LAGB: laparoscopic adjustable gastric banding; LGCP: laparoscopic greater curve plication; LSG: laparoscopic sleeve gastrectomy; %TBWL: percentage of total body weight loss.

Treatment hierarchies for weight loss efficacy were calculated using P-scores (the frequentist equivalent to SUCRA), where a value closer to 1 indicated a higher probability of being the most effective intervention for maximizing %TBWL. LSG was ranked as the most effective treatment (P-score = 0.988), followed by LGCP (0.812) and ESG (0.600). The least effective interventions were LAGB (0.333), HIDLT (0.179), and IGB (0.089) (Fig. 8).

### Inconsistency and publication bias

**Figure 9:**
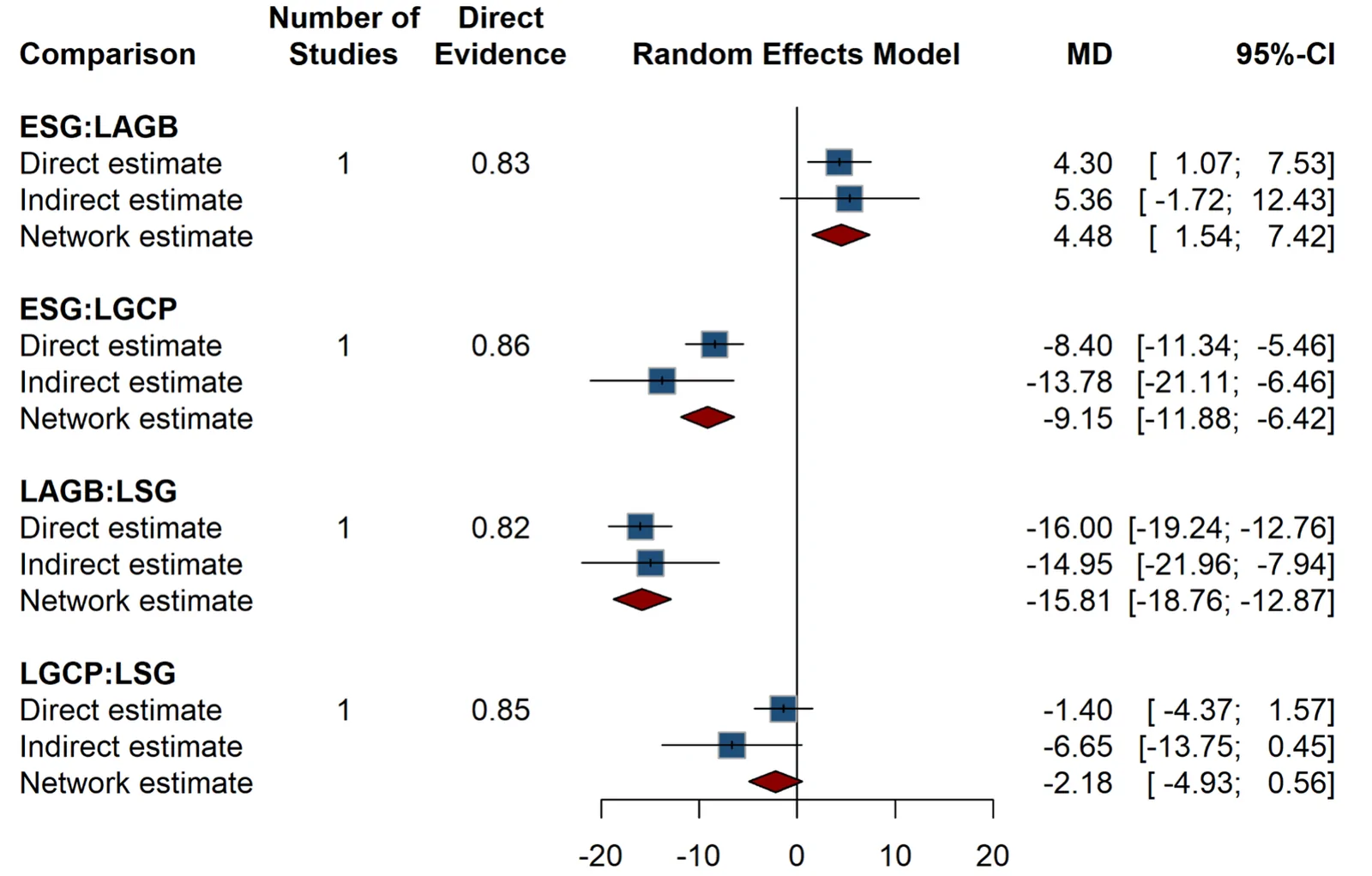
Node-splitting analysis forest plot comparing direct, indirect, and network estimates to assess local inconsistency within the network. ESG: endoscopic sleeve gastroplasty; LAGB: laparoscopic adjustable gastric banding; LGCP: laparoscopic greater curve plication; LSG: laparoscopic sleeve gastrectomy; MD: mean difference; CI: confidence interval.

Global inconsistency within the network was assessed using the design-by-treatment interaction model, which revealed a statistically significant inconsistency across the entire network (Q = 10.41, P = 0.0055). To isolate the source of this inconsistency, a local node splitting analysis was performed (Fig. 9). The node-splitting approach confirmed that the inconsistency was not driven by significant divergence between direct and indirect estimates for key comparisons, such as ESG vs. LAGB (P = 0.79) or ESG vs. LGCP (P = 0.18), suggesting that the network estimates remained internally valid despite the global variation.

**Figure 10:**
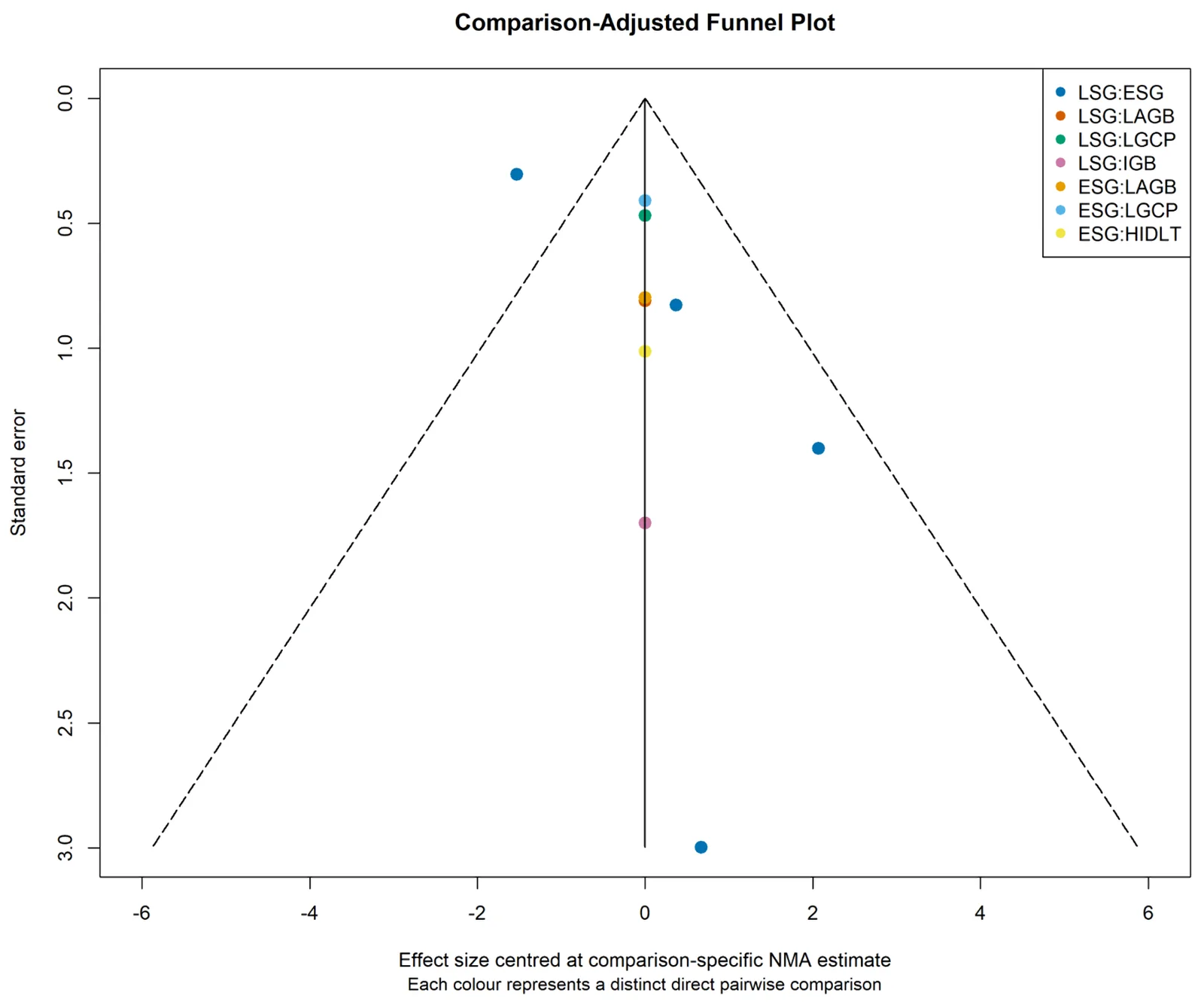
Comparison-adjusted funnel plot assessing publication bias and small-study effects across the network. ESG: endoscopic sleeve gastroplasty; HIDLT: high-intensity diet and lifestyle therapy; IGB: intragastric balloon; LAGB: laparoscopic adjustable gastric banding; LGCP: laparoscopic greater curve plication; LSG: laparoscopic sleeve gastrectomy.

Potential publication bias and small study effects were visually evaluated using a comparison-adjusted funnel plot (Fig. 10). The funnel plot demonstrated relative symmetry around the zero line of the comparison-specific NMA estimates, suggesting the absence of severe publication bias. A formal Egger’s regression test on the direct ESG vs. LSG comparisons did not yield significant evidence of funnel plot asymmetry (*t* = –2.27, P = 0.150); however, this statistical test was underpowered because of the limited number of studies (k < 10) directly comparing these two modalities.

## Discussion

This systematic review and NMA evaluated the comparative effectiveness and safety of EBTs versus conventional bariatric surgery and intensive medical management. Synthesizing data from 17 studies encompassing diverse clinical settings and large-scale national registries, the findings delineated distinct clinical profiles for these modalities. While LSG remains the gold standard for maximizing %TBWL at 12 months, ESG is a highly viable, anatomically preserving alternative that bridges the therapeutic gap between lifestyle modifications and irreversible surgery.

Pairwise analysis demonstrated that LSG achieved a statistically significant, superior weight-loss outcome compared to ESG (MD: –10.53% TBWL, P < 0.0001), a finding further corroborated by the NMA, which ranked LSG as the most effective intervention (P-score = 0.988). The superior metabolic efficacy of LSG is biologically plausible; surgical resection fundamentally alters gastric neurohormonal signaling, precipitating reductions in orexigenic hormones such as ghrelin, which is not fully replicated by the transmural suturing of ESG [4, 7]. However, ESG still achieved robust and clinically meaningful weight loss (ranking third in the network, ahead of laparoscopic gastric banding and IGBs), establishing it as a highly effective intervention for patients who decline or do not qualify for surgical intervention [1, 10].

A critical finding of this study pertains to the safety profile of these procedures, as the meta-analysis of SAEs revealed no statistically significant difference between ESG and LSG (RR = 1.30, P = 0.076), challenging the prevailing assumption that EBTs are universally safer than conventional surgery. Because this comparison was graded as very-low certainty under GRADE, due to residual confounding, imprecision (the 95% CI crossed the null), and the dominance of registry data, the absence of a statistically significant difference should be interpreted as inconclusive rather than as evidence of equivalent safety. This nuance is illustrated by the MBSAQIP analysis of Gudur et al [25], in which major adverse-event rates were indeed comparable between the two procedures (1.4% after ESG vs. 1.1% after LSG; P > 0.05); however, ESG was associated with significantly higher 30-day readmission (3.8% vs. 2.6%), reoperation (1.4% vs. 0.8%), and reintervention (2.8% vs. 0.7%) rates (all P < 0.05). Real-world registry data partly reflect the learning curve associated with emerging endoscopic technologies and underscore that ESG, while incisionless, is not entirely benign and carries specific risks such as perigastric fluid collections and bleeding [5, 9]. The risk of *de novo* GERD, a concerning complication of LSG, is lower after ESG; Dankar et al [28] reported new-onset GERD in 4.3% of ESG patients compared with 20% after LSG (P = 0.004), alongside a shorter hospital stay (9.14 vs. 27.77 h; P < 0.001) [6, 8]. For patients with pre-existing GERD or those highly averse to the surgical morbidity of gastrectomy, ESG remains a strategically advantageous option, provided the higher likelihood of unplanned reintervention is discussed during counselling.

In addition, the findings clarify the declining role of legacy endoscopic interventions, as IGB ranked lowest in the network for weight loss efficacy (P-score = 0.089). Combined with recent literature indicating decreasing utilization trends and high rates of early non-operative reintervention due to intolerance, the data suggest that IGBs are being supplanted by more durable procedures such as ESG and highly effective novel pharmacotherapies (e.g., glucagon-like peptide-1 (GLP-1) receptor agonists) [3]. EBTs are rapidly evolving, and future paradigms will involve EBTs combined synergistically with anti-obesity medications, offering a step-up approach to obesity management [2].

The network was restricted to sleeve-based and restrictive modalities and did not incorporate RYGB. As a partly malabsorptive procedure, RYGB achieves weight loss comparable to or exceeding that of LSG, with superior and more durable resolution of type 2 diabetes and gastroesophageal reflux, albeit at the cost of a more complex anatomy and a higher long-term burden of micronutrient deficiency and internal-hernia risk [2]. With respect to nutritional prognosis, the two sleeve procedures differ mechanistically in ways this synthesis could not directly quantify. LSG is a permanently restrictive operation that reduces gastric capacity and diminishes gastric acid and intrinsic factor production, mechanisms that predispose to micronutrient malabsorption and underpin the routine recommendation of long-term supplementation and biochemical monitoring. ESG, by preserving gastric continuity and the native absorptive surface, would be expected to carry a lower risk of clinically significant malnutrition; consistent with this, severe malnutrition formed part of the adverse-event composite that favored ESG in Osman et al [38]. However, none of the included studies reported standardized long-term micronutrient outcomes, and a formal comparison of nutritional prognosis was therefore not feasible, an evidence gap that prospective studies capturing micronutrient trajectories and lean-mass preservation should address.

The primary limitation of this study is the reliance on observational and registry data, as no head-to-head RCTs directly comparing ESG and LSG were available, resulting in a very low certainty of evidence grading under the GRADE framework, driven by residual confounding and high attrition rates in several longitudinal cohorts. Furthermore, a significant global inconsistency was detected within the network (P = 0.0055), stemming from heterogeneous perioperative dietary protocols, varying baseline BMIs, and the inclusion of distinct healthcare systems. Reporting of baseline anthropometric characteristics was inconsistent across the evidence base, which limited a fully quantitative appraisal of transitivity; notably, the propensity-matched analysis by Osman et al [38] balanced treatment groups on age, sex, race, and comorbidities but not on baseline BMI, leaving open the possibility of residual imbalance in that cohort. In addition, long-term nutritional and micronutrient outcomes were inconsistently reported across the included studies and could not be pooled, precluding any firm conclusion regarding the comparative nutritional prognosis of the two procedures. However, local node-splitting analyses confirmed the alignment between direct and indirect evidence, supporting the overall validity of our network estimates.

### Conclusions

This NMA establishes that while LSG maintains superiority in weight-loss efficacy, ESG is a clinically effective, and less invasive alternative that outperforms legacy endoscopic devices and lifestyle modification. Given the very-low certainty of the safety evidence, ESG cannot yet be declared as safe as LSG; its safety profile appears broadly comparable but requires confirmation in adequately powered randomized trials.

## Supplementary Material

Suppl 1 PRISMA 2020 checklist.

## Data Availability

All data analysed are included in this article and its supplementary files. The search strategies and extraction forms are available from the corresponding author upon request.

## Abbreviations

%EWL: percentage of excess weight loss
%TBWL: percentage of total body weight loss
BMI: body mass index
CENTRAL: Cochrane Central Register of Controlled Trials
CI: confidence interval
DL: DerSimonian–Laird
EBTs: endoscopic bariatric therapies
ESG: endoscopic sleeve gastroplasty
GERD: gastroesophageal reflux disease
GLP-1: glucagon-like peptide-1
GRADE: Grading of Recommendations Assessment, Development, and Evaluation
HIDLT: high-intensity diet and lifestyle therapy
IGB: intragastric balloon
IRR: inter-rater reliability
LAGB: laparoscopic adjustable gastric banding
LGCP: laparoscopic greater curve plication
LSG: laparoscopic sleeve gastrectomy
MBSAQIP: Metabolic and Bariatric Surgery Accreditation and Quality Improvement Program
MD: mean difference
MeSH: medical subject headings
NMA: network meta-analysis
PICO: Population, Intervention, Comparator, Outcomes
PRISMA: Preferred Reporting Items for Systematic Reviews and Meta-Analyses
PROSPERO: International Prospective Register of Systematic Reviews
RCTs: randomized controlled trials
RE: random-effects
RoB: risk of bias
ROBINS-I: Risk of Bias in Nonrandomized Studies of Interventions
RR: risk ratio
RYGB: Roux-en-Y gastric bypass
SAEs: serious adverse events
SUCRA: surface under the cumulative ranking curve
T2DM: type 2 diabetes mellitus
TSA: trial sequential analysis
WHO-ICTRP: World Health Organization International Clinical Trials Registry Platform
